# Disability Inclusion in Corporate Supplier Diversity Initiatives

**DOI:** 10.1007/s10926-024-10190-2

**Published:** 2024-04-05

**Authors:** Nanette Goodman, Fatma Altunkol Wise, Fitore Hyseni, Lauren Gilbert, Peter Blanck

**Affiliations:** 1 Burton Blatt Institute, Syracuse University, Syracuse, NY USA; 2 Rutgers University, New Brunswick, NJ USA

**Keywords:** Supplier diversity, Disability inclusion, Disability-owned businesses, Corporate social responsibility

## Abstract

**Purpose:**

Since the 1960s, federal and state governments and private-sector companies have used supplier diversity initiatives to ensure their supply chains include businesses owned by traditionally economically disadvantaged or underrepresented groups. Originally concentrated on racial and ethnic minority groups, programs have expanded to include businesses owned by women, veterans, LGBTQ+ individuals, and, in some cases, people with disabilities. This study investigates the extent to which disability is included in supplier diversity initiatives of Fortune 500 companies.

**Methods:**

This paper uses a novel data set created by the authors with information on supplier diversity initiatives and Disability, Equity, and Inclusion (DEI) statements in Fortune 500 companies extracted from public sources. This information is combined with data from Compustat, a corporate financial database published by Standard and Poor’s and additional variables from other sources.

**Results:**

75% of the Fortune 500 companies have supplier diversity programs that express a commitment to diversity yet only 49% of those with such programs include disability-owned businesses (38% of all Fortune 500 companies). Among the largest 100 companies, 89% had supplier diversity programs that included disability, almost 6 times the rate Ball et al. reported in 2005. This study finds disability inclusion varies significantly by company size, industry, and whether the company is a government contractor.

**Conclusion:**

Despite the growth in disability inclusion, the absence of disability as a diversity category in regulations mandating supplier diversity initiatives for government contractors impacts disability inclusion. If we want to align our supplier diversity programs with the Americans with Disabilities Act, the first step is to address the issue in the Small Business Administration and federal contracting requirements.

## Background

Supplier diversity initiatives are increasingly becoming a focal point for many organizations as they seek to promote diversity and inclusion in their supply chains. These initiatives involve actively seeking out and engaging with suppliers who are owned by individuals from traditionally underrepresented groups such as women, racial and ethnic minority groups, lesbian, gay, bisexual, transgender and queer (LGBTQ+) individuals, and in some cases, people with disabilities. However, little is known about the extent to which disability-owned businesses are included in these initiatives.

In 2005, Ball et al. examined publicly available data from the top 100 companies listed in Fortune Magazine’s 2003 compilation of the nation’s 500 most profitable companies [1]. The study found 15% of these companies’ supplier diversity policies included disability in their definition of diversity. Since then, a lot has changed. In 2008, Congress passed the ADA Amendments Act (ADAAA) which broadened the definition of disability from the language used in 1990 Americans with Disabilities Act. In 2010, The US Business Leadership Network (renamed Disability:IN in 2018) a non-profit organization, created a Disability-Owned Business Enterprise (DOBE) certification and has been working with over 500 leading companies to expand opportunities for DOBEs. Since 2014, companies have increased their focus on disability inclusion in response to new rules regarding the implementation of Section 503 of the Rehabilitation Act [2]. Section 503, as amended, encourages federal contractors and subcontractors to aim for a workforce in which 7% are individuals with disabilities and to track progress toward reaching this “aspirational goal.” Despite these developments, little research since Ball et al. [1] has studied the inclusion of disability in supplier diversity programs.

A relatively robust literature has explored the motivations to implement a supplier diversity program, their economic impact, the benefits associated with such initiatives for corporations and communities, and the reliability of predictors determining program effectiveness [3–9]. However, most of this research neglects the inclusion of disability within their scope of what is defined as diversity.

Studies that do include disability, highlight the paucity of available information. In a review of Corporate Social Responsibility Plans, Gould et al. found that reports lack specific detail about accomplishments or contracting processes, typically including only a list of the diverse groups considered during the contracting process and the national groups that certify and identify credible contractors [10].

When data are available, they indicate that disability-owned businesses receive an exceptionally small share of spending on diverse suppliers. A recent survey of 466 companies of varying sizes, conducted by Supplier.io found that among companies incorporating disability-owned businesses into their supplier diversity programs, the average spending allocated to DOBEs is below 0.1% [11]. Meta, one of the few companies that publicly reports spending on DOBEs, reports that, of the $1.26 billion spent with diverse suppliers in 2021, less than 1% went to DOBEs [12].

Disability is a relatively new category for most supplier diversity programs. Disability:IN, the only certifier of DOBEs (although other agencies certify Service-disabled Veterans—a subset of DOBEs) launched in 2010 and currently has 600 certified DOBEs [13]. In comparison, the National Minority Supplier Development Council (NMSDC) began in 1972 and reported 15,058 certified minority owned business enterprises (MBE) in 2021 [14].

A significant amount of room for growth exists. National Disability Institute estimated there were 1.8 million small businesses owned by people with disabilities in 2019 [15].The data provide limited information on the size and type of businesses. But they highlight the prevalence of disability-owned business and the unrealized opportunity for increasing the number of certified DOBEs and including more of these small businesses in supplier diversity programs.

### What Is a Supplier Diversity Initiative?

Supplier diversity initiatives are a proactive approach to develop a more inclusive base of suppliers when procuring goods and services often setting “diversity spend” goals to measure their progress. The diversity spend goals vary by company but average around 8–15% of total procurement [11, 16, 17]. Companies vary in the extent to which they publicly report their diversity spend either in total or by each diversity category.

Many supplier diversity programs require certification to validate a company’s ownership status. Certification can be obtained through third-party organizations that verify and authenticate diverse ownership. Examples of certifying organizations include the NMSDC, Women’s Business Enterprise National Council (WBENC), National LGBT Chamber of Commerce (NGLCC), and Disability:IN.

To be considered a diverse supplier, a business must be at least 51% owned and operated by a historically underrepresented or underserved group. Companies tend to include MBEs, women-owned business enterprises (WBEs), service-disabled veteran-owned small businesses (SDVOSB) or veteran-owned small businesses (VOSB), and some include LGBTQ+ owned small businesses and DOBEs.

In addition to setting goals, supplier diversity programs actively seek out diverse suppliers and mentor them through the process. Because buyers are spread throughout different departments in large companies, larger companies tend to have a small, centralized supplier diversity team that works with buyers to identify opportunities to include diverse suppliers in procuring a wide range of goods and services.

### Motivation for Supplier Diversity Programs

Companies with supplier diversity programs specify, either through surveys or on their supplier diversity webpage, that the rationale behind implementing such programs include: government mandates; a general commitment to diversity and inclusion; corporate social responsibility, including a commitment to leveling the playing field for diverse suppliers and enhancing the economic well-being of underserved communities; and a desire for their suppliers to reflect the community to bolster brand image and increase innovation to better serve customers. As we describe in this section, all these motivations argue for the inclusion of disability in supplier diversity programs.

#### Government Mandates

The origins of the early supplier diversity program in U.S. companies can be attributed to the Federal Government’s 8(a) program and related legislation defining the authority of the Small Business Administration [5, 6]. The Small Business Act of 1958, amended in 1978 and several times (Public Law 95-907), created the Section 8(a) program which provides businesses that are at least 51% owned, managed, and controlled by one or more “socially and economically disadvantaged” individuals with access to federal contracting preferences in the form of set-aside and sole-source awards [18].

In addition to influencing the growth of supplier diversity programs by providing a model for private-sector companies to follow, the Small Business Act and its subsequent amendments create a strong incentive for companies to develop these initiatives by stating that prime contractors on federal contracts over $500,000 (or $1,000,000 in the case of construction) “must agree in the contract that small business, veteran-owned small business, service-disabled veteran-owned small business, HUBZone small business, small disadvantaged business, and women-owned small business concerns will have the maximum practicable opportunity to participate in contract performance consistent with its efficient performance.” [19] Disability-owned small businesses are not included in this list.

This clearly motivates some companies. TD Synnex (Ranked 117 on the 2021 Fortune 500 list), for example, specifies on its supplier diversity webpage:

SYNNEX is committed to providing programs and services to help the small-business reseller succeed in this industry. SYNNEX’ Diversity Alliance Program is a community for our diversity partners, system integrators, and vendors that allows each of them to provide technology products for the government while fulfilling government targeted set-aside goals [19].

In 1978, the SBA legislation established a basic definition of *socially disadvantaged individuals*, which included those who have been “subjected to racial or ethnic prejudice or cultural bias because of their identity as a member of a group without regard to their individual qualities” and specified that “Black Americans, Hispanic Americans, Native Americans, and other minorities” are socially disadvantaged [20]. The Minority Small Business and Capital Ownership Development Program—commonly known as the “8(a) Program”—provides participating small businesses with training, technical assistance, and contracting opportunities in the form of set-aside and sole-source awards. Until July 2023, under the 8(a) program members of certain racial and ethnic groups were presumed to be disadvantaged, and other individuals could go through a process to prove personal disadvantage by a preponderance of the evidence. A ruling in Ultima Servs. Corp. v. Dep’t of Ag, barred SBA from using the presumption of social disadvantage to administer the 8(a) Program [21].

A 2015 report from the Congressional Research Services notes that people with disabilities were not presumed to be disadvantaged for the 8(a) program. Furthermore, the report suggests that “there are separate contracting programs” for people with disabilities, in reference to the AbilityOne program. In doing so, the report conflates DOBEs (*owned, operated, and controlled* by people with disabilities) with the AbilityOne program—non-profit organizations (usually led by non-disabled people) that employ people with disabilities often at low wages receiving federal contracts as part of the Javits-Wagner-O’Day Act [22]. This misunderstanding is not unique to this report. In related research that is forthcoming, we have found several state supplier diversity programs have difficulty with the notion that these two types of entities are distinct and that people with disabilities are capable of owning a business.

Other federal contracting opportunities and set-aside programs presume that business owners who are women or service-disabled veterans are disadvantaged (i.e., the SBA Women-Owned Small Business (WOSB) Federal Contract program, and the Vets First Contracting Program-P.L. 109-46.). In contrast, no contracting program presumes business owners with disabilities are socially and economically disadvantaged despite language in the Americans with Disabilities Act to the contrary: “The Congress finds that […] census data, national polls, and other studies have documented that people with disabilities, as a group, occupy an inferior status in our society, and are severely disadvantaged socially, vocationally, economically, and educationally;” and … “the Nation’s proper goals regarding individuals with disabilities are to assure equality of opportunity, full participation, independent living, and economic self-sufficiency for such individuals.” [23]

#### Levels the Playing Field for Diverse Suppliers and Helps Grow Small, Disadvantaged Businesses

Marginalized groups may have less access to information, financial capital, and social capital, thus making it more difficult to compete for contracts. Supplier diversity initiatives represent a way around traditional networks that can often serve as a barrier to entry for firms owned by people from marginalized groups [24]. The supplier diversity website of Walmart (Ranked 1 in the 2021 Fortune 500 list) says, “*This program provides companies owned and operated by minorities, women, veterans, members of the lesbian, gay, bisexual and transgender (LGBT) community, and people with disabilities equal footing to effectively work with us while at the same time, growing their own business.*” [25]

The need to level the playing field applies equally to people with disabilities. The societal barriers that effectively keep people with disabilities from participating in the labor force through salaried employment also affect their efforts to become successful business owners [26, 27]. Entrepreneurs with disabilities report that they face pervasive ableism, are often required to overcome low expectations, have limited access to traditional financing channels and to accessible technologies, and feel excluded from business networking opportunities [15, 26]. People with disabilities have lower levels of social capital, which affects access to financial capital, employment outcomes, and starting wages [28, 29], and health [30]. Extant research indicates that high quality entrepreneurship programs can effectively help entrepreneurs with disabilities increase their self-efficacy and overcome traditional barriers [31]. As a result, companies providing additional support for their diverse suppliers are in a unique position to mitigate entrenched challenges for entrepreneurs with disabilities.

#### Enhances the Economic Well-Being of the Community

By providing opportunities to diverse suppliers, companies help promote economic development of historically underrepresented communities [3, 32]. A study by the NMSDC found that certified MBEs generated $482 billion in economic output and supports 1.8 million jobs [14]. The Billion Dollar Roundtable (BDR), a corporate advocacy organization that promotes companies that have spent at least $1 billion annually with diverse businesses, reports their 32 member companies generated $194 billion in total economic impact and created 1.3 million jobs in 2021 [33].

CVS Health (Ranked 4 on the 2021 Fortune 500 list) highlights this goal on their supplier diversity webpage,

We are working to create jobs and increase economic opportunities for the people and local businesses in the communities we serve [34]

This motivation can be applied to DOBEs as well. Disability:IN reports that certified DOBEs had a total economic impact of $32.3 billion and supported 6,414 jobs [35]. These numbers are supported by other analysis which indicates that the contracts provided by BDR companies to DOBEs created or sustained 4,608 jobs [33]. Based on Disability:IN analysis, DOBEs employ people with disabilities at a rate 10 times that of non-DOBEs, addressing the persistent lack of employment opportunities in the disability community [35].

#### Reflects the Diversity of Their Customers

Many companies state on their supplier diversity webpage that the purpose of their program is to reflect the diversity of their community. This broad statement addresses ethical and economic considerations. First, it demonstrates that the company’s commitment to DEI is an important aspect of corporate social responsibility [36]. Second, it can enhance the company’s reputation by highlighting their commitment to diversity and inclusion, which can help to attract customers and investors who share these values. Including DOBEs in supplier diversity initiatives has been shown to enhance a corporation’s image to the larger community [37]. As demographics change, corporations use their supplier diversity programs as an avenue to develop closer and more sustainable links with the minority populations who are actual or potential customers [6]. UPS (Ranked 35 on the 2021 Fortune 500 list) reflects the business case:

By working with suppliers who reflect the markets we serve, we see a wealth of benefits: it helps build customer loyalty, contributes to economic development of communities, and provides the expertise and innovation we need to outperform the competition. That is why we are committed to building a diverse and inclusive supply chain [38].

With an estimated 61 million Americans living with a disability [39], the disability community is a sizeable market of customers. In 2018, researchers estimated the total after-tax disposable income for working-age people with disabilities is about $490 billion, similar to that of other significant market segments, such as African Americans ($501 billion) and Hispanics ($582 billion) [40].

#### Fuels Innovation

Through diverse suppliers, companies gain access to new ideas and perspectives that align with the needs of customers, which help drive innovation and growth [41, 42]. AmerisourceBergen (Ranked 8 in the 2021 Fortune 500 list) describes the motivation for their commitment as follows:

Working with these diverse suppliers helps us source the highest quality products and services at the most competitive prices, but to us it’s much more than that. Our commitment to supplier diversity comes straight from the top of our organization and it’s integrated throughout all of our applicable businesses. And through our growing program, we’re able to directly support companies that are addressing the unique needs of underserved communities and fuel innovation where it’s needed most [43].

Sprouts (Ranked 437 in the 2021 Fortune 500) also focuses on the business case by saying the following:

We[…] believe that sourcing products from diverse suppliers such as minority- and women-owned businesses not only supports the economic well-being of the local communities we serve, but also builds on our strategy of winning with our target customer by providing them with innovative grocery options from emerging brands [44].

Companies are increasingly recognizing the need for user-centered and inclusive design approaches. By considering the needs of people with disabilities, designers can create products and environments that are accessible to all, fostering inclusivity and equal opportunities. Entrepreneurs with disabilities are in a unique position to provide these innovations. As inhabitants of a world that can be inaccessible to them, individuals with disabilities must find alternative ways to do the things they want and need to do in life. Their unique perspectives, experiences, and needs are invaluable in developing inclusive and accessible solutions that may be relevant not just for people with disabilities, but for the broader population [45].

#### Commitment to Disability Inclusion

Including disability in supplier diversity programs is often an indication of a company’s broader commitment to disability inclusion that may include disability hiring and retention initiatives and cultural shifts in the organizations. This commitment is reflected in its Diversity, Equity, and Inclusion (DEI) statement.

Given the value of including disability-owned businesses in supply chain diversity programs and the alignment with the stated goals of the initiatives, we sought to answer the following research questions:

1. Among the Fortune 500 companies, how many include disability in their supplier diversity programs?
2. What company characteristics predict inclusion of disability?
3. To what extent are companies driven by government policy affecting federal contractors?

## Methods

### Study Design and Data

We created a novel data set with information on supplier diversity initiatives for each of the 2021 Fortune 500 companies. To determine whether a company had a supplier diversity initiative and whether it included disability, we employed a two-step process. Initially, we used the Google search engine to locate the supplier diversity website of each of the 500 companies. Most websites listed the specific groups included in their initiative or provided a list of certifications accepted. If a website explicitly mentioned disability or indicated acceptance of the Disability:IN certification, we coded the company as “Disability Inclusive.” Sites that mentioned specific types of disability groups such as disabled veterans or “physically challenged” but did not include all disabilities were coded as “Partial Disability Inclusive.” Notably, several companies listed “sheltered workshops,” or “qualified non-profit agencies for the blind and other severely disabled enterprises” in lieu of disability-owned businesses. These were coded as “Disability Absent” unless they also included another disability group. In cases where we were unable to locate the supplier diversity website or if the website did not specify the diverse groups covered, we took the relevant information from the companies’ Environmental, Social, and Governance (ESG) reports. If we found no evidence of a supplier diversity website and no mention of supplier diversity in the ESG report, we coded the company as not having a supplier diversity initiative (No program).

A similar process was used to code the inclusion of disability in DEI statements. Using Google search, we located either the DEI page within the company webpage or as part of the company-specific careers webpage. Considering the importance of signaling a welcoming and inclusive environment to current and future employees as well as customers, we only considered clear DEI statements available in the main company webpages but not any statements that were not publicly available or included in mandatory company reports. Those companies that had a public-facing DEI statement were coded as ‘Have DEI Statement’ and those that did not were coded as ‘No DEI Statement’. Among those companies that had DEI statements, we created detailed subcategories. Companies that explicitly stated that they are inclusive towards people with disabilities were coded as “DEI Disability Inclusive.” Those companies that had DEI statements but did not list any groups were coded as “DEI Ambiguous.” Finally, those companies that listed underrepresented groups in their statement but did not include disability were coded as “DEI Disability Absent.”

We merged the novel data set with 2016 and 2021 data from Compustat, a product of Standard & Poor’s (S&P) Global Market Intelligence that provides extensive historical and current financial data on publicly traded companies. Of the Fortune 500 companies, 479 are publicly traded and included in the Compustat database. In the few cases where 2016 or 2021 data were not available, we used the most recent available year.

Based on existing literature that suggests that democratic-leaning voters care more about Corporate Social Responsibility (CSR), [46–48], we incorporated state-level data from the Pew Research Center Religious Landscape Study from 2014 to add a measure of the party leaning of adults in the state where the company is headquartered. We coded whether the company was a federal contractor based on data from General Services Administration Contractor Directory [49], U.S. Small Business Administration, Directory of Federal Government Prime Contractors with Subcontracting Plans [50], and System for Award Management Fiscal Year 2021 Top 100 Contractors Report [51].

### Study Measures

#### Primary Outcome Measure

##### Disability Inclusion in Supplier Diversity

This variable has four possible values: (1) No Supplier Diversity Program, (2) Disability Absent (Company has program, but does not include disability), (3) Partial Disability Inclusion (such as service-disabled veteran or only physical disabilities) and (4) Disability Inclusive (Program includes all disability). Based on publicly available data, 38.0% of the Fortune 500 companies have a Disability Inclusive Supplier diversity program. Additionally, 14.8% partially include disability in their supplier diversity programs meaning that they specify a subset of the disability community like service-disabled veterans or “physically challenged” as eligible (Table 1).

**Table 1:** Descriptive statistics of the sample

Variables	Pct/Mean	SE (for continuous variables)
Inclusion of disability in supplier diversity programs (*n*=500)		
No program	24.8%	
Disability absent	22.4%	
Partial disability inclusion	14.8%	
Disability inclusive	38.0%	
Inclusion of disability in corporate DEI statements (*n*=500)		
No DEI policy	7.0%	
DEI disability absent	11.4%	
DEI disability ambiguous	18.4%	
DEI disability inclusive	63.2%	
Type of industry (*n*=479)		
Manufacturing	34.5%	
Finance, insurance, and real estate	17.1%	
Transportation, communications, electric, gas, and sanitary services	12.5%	
Retail trade	11.9%	
Services	11.1%	
Wholesale trade	6.9%	
Mining	2.5%	
Construction	2.3%	
Other	1.2%	
Other company-level control variables (*n*=479)		
Number of employees	63,585	6,828
Total revenue (in $ thousands)	32,294	2,554
Pretax profit margin	14.23	0.61
Percent Republican leaning in State	35.0%	
Government contractor	32.8%	
New Fortune 500 list	34.7%	
Pct change in employees	26.0%	4.8%
Pct change in revenues	65.7%	8.6%
Change in profit margin	3.2	1.2

*Note:* The descriptive statistics for the first two variables are for the Full Sample=500 and the rest are for the Compustat Sample=479. Represented here is the mean for continuous variables or percent for categorical variables. Category other in type of industry includes Agriculture, Forestry, and Fishing, and Public Administration

#### Company Characteristics

##### Fortune 500 Rank

**Fig. 1:**
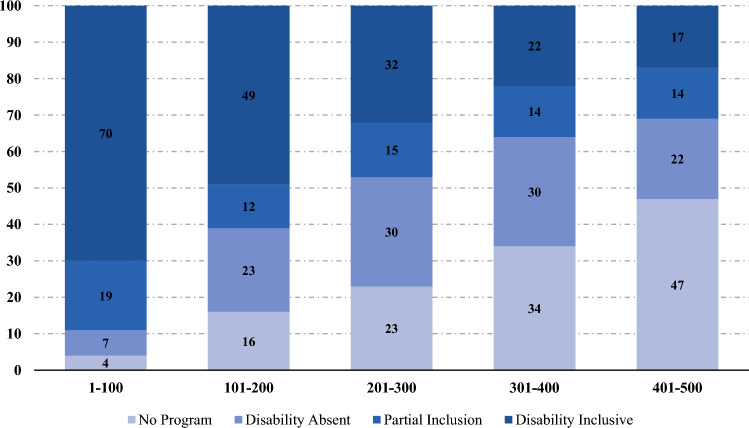
Inclusion of disability in supplier diversity programs by Fortune 500 Rank. (*Note*: Full sample=500)

Based on the 2021 list published by Fortune Magazine, the rank is based on annual revenues where a rank of 1 represents the largest company and 500 denotes smallest of the top 500. Annual revenues ranged from $6.2 billion for the smallest company to $560 billion for the largest. For ease of display in Fig. 1, we divided the Companies into five groups of 100 based on their rank.

**Number of employees**, a Compustat variable, represents the number of company workers as reported to shareholders. This is reported by some firms as an average number of employees and by some as the number of employees at year-end. Compustat does not differentiate between these bases of reporting. If both are given, the year-end figure is used. The average number of employees reported by Fortune 500 companies is 63,585.

**Total revenue,** a Compustat variable, represents the gross income received from all divisions of the company. Average total revenue is $32,294,000.

**Pretax Profit Margin** is computed using Compustat data by dividing Pretax Income (which includes fiscal year end operating and non-operating income before taxes and minority interest) by Net Sales. The average pre-tax profit margin is 14.23.

##### Type of Industry

We used Standard Industrial Classification (SIC) codes to categorize businesses based on their primary economic activities. Four digit SIC codes are organized into ten divisions representing broad sectors of the economy (A-J). Each division is then divided into Major Groups and then Specific types of businesses providing a hierarchical structure for industry classification (US Department of Labor, n.d.). Our analysis is based on the ten SIC divisions. However, because a two SIC divisions “Agriculture, Forestry, and Fishing” and “Public Administration” had fewer than 10 companies, we collapsed these into an “other” category. More than one-third (34.5%) of the companies in our analysis are in Manufacturing; 17.1% in Finance, Insurance, And Real Estate; 12.5% in Transportation, Communications, Electric, Gas, And Sanitary Services", 11.9% in Retail Trade, 11.1% in Services; 6.9% in Wholesale Trade; and under 3% in Mining and Construction.”

##### Percent Change in Employees, Revenues, and Pre-tax Profit Margin

Using two different years of Compustat data, these variables are calculated by dividing the difference in the values (as defined above) in 2021 and 2016 by the 2016 values. The average percent change in employees, revenues, and pre-tax profit margin for our sample was 26.0%, 65.7%, and 3.2%, respectively. Twenty-one companies were dropped from the logit analysis because of missing Compustat data needed to compute these variables. We refer to this sample as the ‘Compustat Sample’ and the full Fortune 500 list as the ‘Full Sample’.

##### Inclusion of Disability in Corporate DEI Statement

Similar to Disability Inclusion in Supplier Diversity, the variable has four values (1) No DEI Policy, (2) Has DEI policy but does not include disability (3) DEI policy is ambiguous about its inclusion of disability and (4) DEI policy includes disability. Almost two-thirds (63.2%) have disability inclusive DEI statements.

#### Other Independent Variables

##### Percent Republican Leaning in State

The variable represents the percentage of the population in the state that identified as Republican or identified as Independent, no preference, or Party other than Republican or Democrat, and report leaning Republican. The mean value for this variable was 35.0%.

##### Government Contractor

This is a binary variable coded 1 if the company is listed on the GSA Contractor Directory, the SBA Directory of Federal Government Prime Contractors with Subcontracting Plans or as a top contractor for any of the agencies reported in the SAM.gov Fiscal Year 2021 Top 100 Contractors Report. About one-third (32.8%) of our sample were reported to be government contractors.

##### New to the List of Fortune 500 Companies Since 2011

This is a binary variable determined by comparing the Fortune 500 lists in 2011 and 2021. It is coded 1 if the company was not on the list in 2011. The variable is used to distinguish between companies that have experienced significant growth and those that have maintained their positions as well-established, large corporations. About one-third (34.7%) of the companies in the Fortune 500 2021 list were new.

### Analyses

We use descriptive statistics to explore the relationship between company characteristics and the four categories of disability inclusion in supplier diversity programs. For categorical variables such as Fortune 500 group, Industry, and Inclusion of disability in Corporate DEI Statement, we developed cross-tabulations and conducted Chi-square tests to determine the presence of significant association between the supplier diversity categories and the other variables.

For continuous variables, we calculated the mean values of each of the company characteristics within each of the supplier diversity categories and conducted one-way analysis-of-variance (ANOVA) to measure the magnitude and associated statistical significance of the correlation.

To identify the relative importance of key predictors of supplier diversity outcomes, we estimated two logit models. Using the same independent variables, the first model estimates the probability of a company having a supplier diversity program. The second, a multinomial logit, estimates the probability of being disability inclusive conditional on having a program.

## Results

### Relationship Between Disability Inclusion in Supplier Diversity Programs, Company Size, Industry, and Disability Inclusion in DEI Statements

#### Company Size

Among the Fortune 500 companies, 375 had a supplier diversity program (75.2%), 38% had fully inclusive supplier diversity programs, 14.8% had partially inclusive programs, and 22.4% had programs that did not include disability (see Fig. 1). Among the largest 100 companies, 89% had supplier diversity programs that included disability, almost 6 times the rate Ball et al. reported in 2005.

Figure 1 shows a linear trend in that among the 100 largest US companies, 70% have a disability inclusive supplier diversity program, compared to only 49% for those ranked 101–300, 32% for those ranked 201–303, 22% for those ranked 301–400, and 17% for those ranked 401–500.

#### Industry Type

**Fig. 2:**
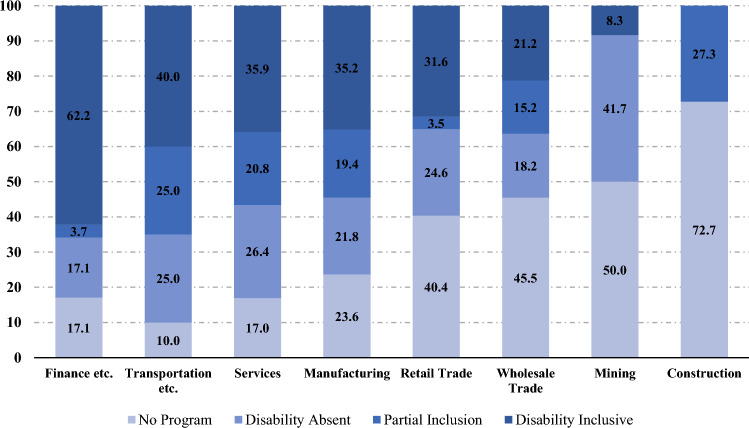
Inclusion of disability in supplier diversity programs by Industry Sector (Percentage). (*Note*: *n*=473. Analysis restricted to Public Companies. Agriculture and Public Administration omitted due to small number of cells)

Disability inclusion in supplier diversity programs is also highly correlated with industry type. Finance, insurance, and real estate industries have the highest number of companies with disability inclusive supplier-diversity programs (62.2%; see Fig. 2) whereas fewer than one-third of Fortune 500 companies in retail trade, wholesale trade, mining, or construction had a fully inclusive supplier diversity program.

#### Relationship Between Disability Inclusion in Supplier Diversity Programs and DEI Statements

**Fig. 3:**
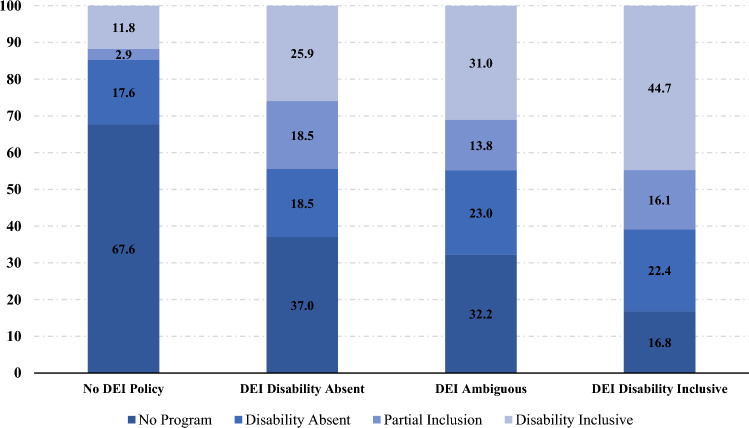
Inclusion of disability in supplier diversity programs by disability inclusion in DEI statements. (*Note*: Full sample=500)

We explored the relationship between supplier diversity programs and the presence of public-facing DEI statements. Figure 3 shows that 25% of companies that have disability inclusive supplier diversity programs but did not fully include disability in their DEI statements. We also see a linear trend such that 75.3% of companies that included disability in their supplier diversity program had a disability inclusive DEI statement compared to only 67.6% of those that included disability partially, 64.3% of those that did not include disability, and 41.1% of those that did not have a supplier diversity program at all. These differences are significant as shown by our pairwise comparison in Table 2 that indicated that having a DEI statement is positively correlated with having a supplier diversity program. Surprisingly, the converse is not true. Excluding disability from the DEI statement is not correlated to excluding it from the supplier diversity program.

**Table 2:** Comparison of company characteristics’ mean values across four categories of supplier diversity inclusion (public companies only)

	No program	Disability absent	Partial inclusion	Disability inclusive	*p*-value
Mean values in 2021					
Fortune 500 Rank	341.71	288.62	238.86	175.93	0.00
Number of employees (in thousands)	27,439	46,255	51,384	102,898	0.00
Total revenue (in $ millions)	$14,096	$18,843	$27,620	$54,149	0.00
Pretax profit margin	11.22	13.27	12.22	17.62	0.00
Percent Republican leaning in State	35.9%	34.9%	35.3%	34.3%	0.09
Government contractor	11.5%	36.5%	59.7%	34.3%	0.00
New to Fortune 500 list	58%	35%	28%	22%	0.00
Changes 2016–2021					
Pct change in employees	39%	23%	17%	23%	0.45
Pct change in revenues	101%	78%	39%	46%	0.04
Change in profit margin	4.95	6.60	−3.33	2.69	0.07

*Notes:* p-value presented here is associated with ANOVA test for continuous independent variables and Chi-squared test for categorical variables

#### Other Company Characteristics

In Table 2, we present mean values of each variable by type of supplier diversity program as well as ANOVA and Chi-square statistic to test the association between our dependent and independent variables. The ANOVA F-value indicates that companies with different levels of inclusion differ on most of the company characteristics.

In particular, the presence of a supplier diversity program and the inclusion of disability within the program is correlated with size, whether measured by Fortune 500 rank, number of employees, or total revenue. Companies with disability-inclusive supplier diversity programs are also more likely to have a higher profit margin. However, this may be associated with industry [52]. Being a government contractor is highly correlated with having a supplier diversity program but the relationship with the inclusiveness of the program is ambiguous. Only 11.5% of companies with no program are government contractors compared with 36.5% of those with disability absent programs, 59.7% with partially inclusive programs, and a 34.3% of disability inclusive programs. Additional correlations show that companies in more Republican leaning states are slightly less likely to have a disability-inclusive program. Fast growing companies (based on measures of New to the Fortune 500 list and Percent change in employees in revenue) are less likely to have a disability inclusive supplier diversity program than more established companies.

The independent effects of these variables are more rigorously tested in the multivariate analysis described in the next section.

### Multivariate Analysis

To estimate the effect of company characteristics on the inclusion of disability in supplier diversity programs, we estimated two models using the same independent variables. The first model (Model 1), a logit, estimates the probability that the company has a supplier diversity program. The second, a multinomial logit (Model 2) estimates the relative probability of the program being partially disability inclusive or fully inclusive compared to disability absent conditional on the company having a supplier diversity program. To further simplify the interpretation of the results, we convert the odds ratio into marginal effects. Specifically, we compute average marginal effects (AMEs) for specific independent variables while keeping the values of other independent variables as they are observed. This helps us show how the probability of having a supplier diversity program or including disability is affected when our independents variables change. We discuss below only the marginal effects of the coefficients that are statistically significant in the main model.^1^

Two strong results emerge from the logit results on having a supplier diversity program (see Table 3). The first is that while being a larger company (based on Fortune 500 rank) increases the likelihood of having such a program, it is mostly being a long-time large company that is associated with it. On average, the probability of having a supplier diversity program for companies that joined the Fortune 500 after 2011 is 10 percentage points lower than for those that joined prior to 2011. Second, being a government contractor has, by far, the largest correlation with having a supplier diversity program. On average, government contractors are 18 percentage points more likely to have a supplier diversity program compared to those that are not government contractors.

**Table 3:** Marginal effects of characteristics related to disability inclusion in supplier diversity programs

	Model 1	Model 2		
Variables	Has supplier diversity program	Does not include disability	Partially includes disability	Fully includes disability
Fortune 500 Rank	***−***0.0005***	0.0009***	0.0000	***−***0.0009***
Conservative	***−***0.5267	0.0350	0.2259	***−***0.2609
number of employees	0.0927**	***−***0.0028	***−***0.0551	0.0579
pre-tax profit Margin	0.0025	***−***0.0033	***−***0.0007	0.0040*
% Change in employees	0.0059	***−***0.0839	0.0333	0.0506
% Change in revenue	***−***0.0021	0.1074*	***−***0.0510	***−***0.0564
Change in profit margin	***−***0.0010	0.0014	***−***0.0007	***−***0.0008
New to the Fortune 500	***−***0.0986***	***−***0.0490	0.0354	0.0136
Govt contractor	0.1770***	0.0180	0.0935**	***−***0.1116**
industry				
Manufacturing	***−***0.0772	0.0394	0.1892***	***−***0.2285**
Transportation, etc.	0.0456	0.0175	0.1967***	***−***0.2142**
Retail trade	***−***0.2085**	0.2647**	***−***0.0135	***−***0.2511**
Services	***−***0.0222	0.0398	0.1777**	***−***0.2175**
Wholesale trade	***−***0.2233**	0.0968	0.1356	***−***0.2324*
Mining	***−***0.1596	0.7664**	***−***0.0630	***−***0.7035**
Construction	***−***0.3108**	***−***0.2336***	0.9370***	***−***0.7034***
Other	***−***0.4008	***−***0.2336***	0.4083	***−***0.1747

*Note*: Model 1 shows the AMEs of having a Supplier Diversity Program for the Compustat Sample (*n*=453). Marginal effects are calculated using estimates from the logit model. Model 2 shows the AMEs of three types supplier diversity programs: those inclusive of disability, those partially inclusive, and those that do not include disability (*n*=339). Marginal effects are calculated using estimates from the multinomial logit model. For industry, the reference category is Finance. Only coefficients for Fortune 500 Rank, New to the Fortune 500, and Government Contractor are statistically significant in the logit model (model 1). Fortune 500 Rank and % Change in Revenue are statistically significant in the multinomial logit model (model 2). ***p<0.01, **p<0.05, *p<0.1

However, while being a government contractor is positively correlated with having a supplier diversity program, it is not correlated with whether the program includes disability. This result suggests that while government policies incentivize having a supplier diversity program, exclusion of disability from those policies leads companies to ignore that aspect of diversity.

We did not include the variable measuring the inclusivity of the DEI statement in the regressions predicting the inclusivity of the supplier diversity programs. They are strongly correlated but this is unlikely to be a causal relationship. Rather, they are both signals of a firm’s underlying commitment to disability inclusion.

## Discussion

The current findings contribute to research exploring understanding and importance of incorporating disability in supplier diversity programs within the private sector. To the best of our knowledge, this empirical study represents the first endeavor to investigate different aspects of disability-inclusive supplier diversity programs and their relationship with organizational characteristics and policies. Our findings reveal a significant increase in the presence of disability-inclusive supplier diversity programs since the last study on the issue conducted in 2005. Furthermore, our research highlights the influence of company size and growth patterns, the connection between disability inclusion in supplier diversity and the presence of disability within a company’s diversity, equity, and inclusion (DEI) statement, and the importance of government regulations.

Finding 1: Increased Adoption of Disability-Inclusive Supplier Diversity Programs: Our analysis suggests a heightened awareness among organizations regarding the importance of including DOBEs in their supplier networks. The results of annual surveys conducted by reputable third-party supplier diversity organizations, such as Supplier.io and Billion Dollar Roundtable, further corroborate this finding.

Finding 2: Company Size and Inclusive Supplier Diversity Programs: Another key finding is the positive association between company size (as measured by revenue) and the likelihood of having an inclusive supplier diversity program that incorporates DOBEs. Larger more established companies, companies that have solid but slower growth rates and have been in the Fortune 500 for more than 10 years, are more likely to have inclusive programs. This may be attributed to the greater resources and capacity available to larger organizations, enabling them to implement and sustain comprehensive supplier diversity programs. Another reason could be that larger companies often have more diverse workforces in terms of demographics, skills, and experiences. The diverse nature of their employees provides a stronger force for implementing inclusive DEI policies. They are also more likely to face public pressure to demonstrate their commitment to diversity and inclusivity. Edmans et al. found similar trends in their research with 58 companies and their DEI practices [53].

Finding 3: Supplier Diversity and DEI Statements: The study reveals a significant positive relationship between disability inclusion within supplier diversity programs and the presence of disability-related language in a company’s broader diversity, equity, and inclusion (DEI) statement. The findings suggest that organizations committed to disability inclusion are more likely to articulate their commitment to disability inclusion across their diversity efforts.

Finding 4: Role of Government Regulations: Our analysis reveals a substantial positive correlation between the presence of government contracts and the adoption of supplier diversity programs. However, we find no effect of government contracts on disability inclusion within supplier diversity programs. This notable finding suggests that the leadership provided by the 8(a) program and the requirement to include diverse subcontractors in the Federal Acquisition Regulations may drive overall supplier diversity efforts. However, because disability is not included as a diversity category, the benefit of government policy does not extend to the disability community.

## Limitations

There are several limitations to this study. First, we focus on the extent to which companies publicly list disability as an included category in their supplier diversity initiatives. Because most companies do not disclose the amount of their “spend” on this category, we are not able to capture whether this inclusion leads to tangible business contracts or is merely rhetoric.

Second, our data analysis captures a single point in time, which limits the ability to assess any potential changes or trends in disability inclusion over a longer duration. Companies may have added or revised their programs since the data collection in 2021, and particularly as the COVID-19 pandemic has waned with more employees returning to the workplace.

Third, our analysis considers the inclusion of disability in the supplier diversity programs of the Fortune 500 companies directly and does not consider the extent to which these companies require DOBE inclusion in their tier 2 contracts. That is, it does not analyze opportunities that arise from larger contracts awarded to prime suppliers. Small disability-owned businesses may find greater opportunities in securing tier 2 contracts. The exclusion of tier 2 contracts may lead to an understatement of the overall significance of disability inclusion in supplier diversity initiatives.

## Conclusion

The inclusion of DOBEs in supplier diversity programs has shown promising growth, primarily driven by large companies. However, more research is needed to understand the factors that motivate companies to include disability in their supplier diversity initiatives and to determine the actual impact of such inclusion on securing contracts for DOBEs.

This research would be particularly timely. Two important federal court rulings may affect the future of supplier diversity programs as it relates to racial classification. A lower court’s ruling in *Ultima Servs. Corp. v. Dep’t of Ag,* barred SBA from using the presumption of social disadvantage to administer the 8(a) Program.

While it appears that companies are currently driven more by their commitment to DEI rather than government mandates, it remains important for disability to be included as a socially and economically disadvantaged group in federal programs on par with race and gender. This would increase the visibility of DOBEs and ensure a consistent and systematic approach towards promoting opportunities for DOBEs. The full implications of the decision in *Ultima Servs. Corp. v. Dep’t of Ag,*, however, have yet to be seen in regard to the SBA’s 8(a) program.

In addition, the recent U.S. Supreme Court Decision *Students for Fair Admissions v. Harvard* (2023) in which the Court struck down college admissions determinations based on affirmative action programs associated with race may have serve to narrow programs in the workplace and ultimately affect the sustainability of programs, such as supplier diversity programs, that may have been initially designed to address racial inequality, but also include disability as one of multiple diversity categories.

The economic impact of including DOBEs in supplier diversity programs is limited by the number of DOBEs. Additional research is necessary to explore the barriers and facilitators to expanding the number of companies certified by Disability:IN. That is, Are potential DOBEs hesitant to get certified? If so, why?

While progress has been made in the inclusion of disability-owned businesses in supplier diversity programs, further research, increased support, and ongoing commitment in a changing legal environment are necessary to overcome existing barriers, sustain growth, and mitigate potential backlash.

## Footnotes

1 We do this because the values of marginal effects are contingent on how the values of the other variables in the model are set. In addition, marginal effects are non-linear functions of the estimated parameters. While they useful in interpreting the results, we are interested in testing the hypothesis about the function of all the coefficients, not just one, and as such rely on p-values from the main models.
